# Social Interest Data as a Proxy for Off-Label Performance-Enhancing Drug Use: Implications and Clinical Considerations

**DOI:** 10.7759/cureus.52011

**Published:** 2024-01-10

**Authors:** Philip A Holubeck, Andrew C Eksi, Kyle Gillett, James O'Hara, Daniel J McGoldrick, Demi R Brown, Alec D McCarthy

**Affiliations:** 1 College of Medicine, University of Nebraska Medical Center, Omaha, USA; 2 Department of Family Medicine, University of Kansas Health System, Olathe, USA; 3 Department of Computer Science, California State University, Seaside, USA; 4 School of Medicine, Creighton University, Omaha, USA; 5 Department of Surgery - Transplant, University of Nebraska Medical Center, Omaha, USA

**Keywords:** exercise sciences, sports medicine, drug abuse, anabolic steroid, exogenous steroids, google trends healthcare, anabolic androgenic steroid, selective androgen receptor modulator

## Abstract

Performance-enhancing drugs (PEDs) can be categorized into various classes based on the physiological mechanism of the compound, with the most popular being anabolic steroids, selective androgen receptor modulators, and growth hormones. Ancillary compounds, such as selective estrogen receptor modulators (SERMs) and selective estrogen receptor degraders, are commonly utilized alongside a PED to counterbalance any potential undesired side effects. With little clinically relevant data to support the use of these ancillary compounds, medical education and evidence-based approaches aimed at monitoring the potential adverse effects of PED use are sparse.This study aims to identify emerging trends in the interest of PEDs and related ancillary compounds, hypothesize the physiological effects of the continued respective behavior, and propose a proxy for use by clinicians to approximate off-label drug use and subsequently modify their practices accordingly. Several significant trends were identified for non-FDA-regulated compounds (i.e., selective androgen receptor modulators such as RAD-140) and off-label indications for FDA-regulated drugs (i.e., SERMs such as tamoxifen). A significant increase in interest regarding selective androgen receptor modulators, mirrored by anecdotal reports in clinical settings and online forums, is coupled with stagnant or decreasing interest in both post-cycle therapies and anabolic steroids. Ultimately, we propose a call to action for utilizing social data and/or prescription data as a proxy for clinicians to better understand trends in these compounds and thus refine their treatment protocols in a concordant manner.

## Introduction

For decades, competitive athletes and bodybuilders have utilized a variety of performance-enhancing drugs (PEDs) to improve strength, body composition, athletic performance, and aesthetic appeal. Several classes of prescription, over-the-counter (OTC), and illegal pharmacological agents are regularly utilized for their anabolic and performance-enhancing abilities, with varying results. Nonetheless, professional and recreational use of PEDs and their ancillaries (post-cycle therapies (PCTs), selective estrogen receptor modulators (SERMs), aromatase inhibitors (AIs), etc.) continues to sustain high levels of public interest [1]. While some studies have sought to quantify interest and the use of PEDs, analyses are typically limited to certain classes of drugs, narrow demographics, and singular case reports. Alternatively, public search data may serve as a valuable tool for analyzing interest in PEDs and ancillary compounds without relying on potentially misleading prescription records [2,3]. Here, the public interest in certain PEDs was ascertained and analyzed using Google Trends (GT). Specific PEDs that were analyzed here included androgenic anabolic steroids (AAS), selective androgen receptor modulators, growth hormones (GHs), designer prohormones, and ancillary compounds.

GT is a publicly available platform allowing users to access normalized, unfiltered search volume data expressed in terms of relative search volume (RSV). The RSV is calculated by standardizing the proportionality of a given search term to gross total searches on a 0-100 scale. The totality of searches is determined by GT based on user-defined parameters, such as temporal period and geographic region; thus, these parameters can be specified to best address each user's area of interest. The time constraints allow for analyses spanning from real-time data to intervals such as the past year, past month, etc. Geographical and regional constraints allow for analyses with parameters to include specific cities, counties, states, countries, or even worldwide analysis. Duplicate searches from the same Google search engine user within a short period of time are excluded from the collected data, and RSV values less than 1% are expressed as 0. GT queries are not case sensitive (i.e., “SARMs” and “sarms” will generate identical RSV values); however, GT queries are character sensitive (i.e., “post cycle therapy” and “post-cycle therapy” will generate different RSV values). Previous studies by other healthcare providers have demonstrated that GT may be an effective tool for predicting trends in participation in vaccination, estimating participation in elective surgical procedures, and measuring social interest in science-related topics [3-5]. These studies suggest that the aggregate volume of search queries can be used to indirectly examine popularity within given temporal and regional parameters. In this study, specifically, RSV was utilized to identify trends in PED interest.

The search terms of interest in this study were included for their ability to be utilized as PEDs. The search terms include AAS, selective androgen receptor modulators, GHs, AIs, and SERMs, which all find clinical use as either hormones or hormone modulators. When used under clinical guidance, these compounds have the theoretical potential to improve the quality of life, anabolic capacity, and self-image of users. Nonetheless, each of these compounds poses inherent risks and has noted side effects that, when used off-label or without clinical guidance, put users at high risk for adverse effects: gynecomastia, cardiovascular disease, hypogonadism, male pattern baldness, testicular atrophy, virilization in women, delayed or interrupted puberty, and hypertension, among many others. Noting that these vast complications are increased with improper use of PEDs, it is crucial to understand the nuances of supplementation in order to strengthen clinical education on their pharmacokinetics, pharmacodynamics, and use protocols.

Although prescription data exists, accessing this data is logistically challenging for individual physicians due to the required payment for such information; further, this data does not account for monitoring off-label or illegal usage. This study alternatively proposes utilizing RSV determined from a user-customized GT search as a proxy for off-label drug use of PEDs. By tracking which drugs are being used, their relative popularity within a recreational context, and understanding their overall temporal and regional trends (i.e., SARMs emerging as the most popular PED), healthcare providers can effectively educate patients on the ramifications of off-label drug use in a manner that matches usage rates within the population, as well as self-educate themselves on specific compounds that are the most popular, and thus relevant, for their practice.

Androgenic anabolic steroids

AAS, a class of synthetic drugs modeled after the male sex hormones testosterone and dihydrotestosterone (DHT), were originally intended to treat various hormonal disorders (i.e., hypogonadism). Despite this, AAS are commonly used illicitly to induce skeletal muscle growth for a gain in overall muscle mass and aesthetic definition. This use is due in part to the lack of tissue specificity and expansive, systemic activation of androgen receptors when these compounds are either injected subcutaneously or ingested orally [4]. Biochemically, AAS function as ligands, passively diffusing through the plasma membrane of their target cell and interacting with androgenic receptors (ARs) in the cellular cytoplasm. Once bound, the ARs enter the nucleus and act as transcription factors, leading to the upregulation of genes designated to control protein synthesis and androgenic hormone production. This produces supraphysiologic quantities of androgens, which induce muscular hypertrophy from increased intracellular anabolic activity. It is the upregulation of androgenic hormone production that leads to systemic consequences regarding the endocrine system, such as increased visceral adipose tissue and stimulation of erythropoiesis [5-7].

In an effort to avoid such deleterious repercussions, individuals who utilize AAS will typically "cycle" their compounds on a schedule of 6-12 weeks of use, followed by a several-month-long interval of abstinence [5]. Despite such measures, it is common for users to experience a range of virilizing effects, including accelerated male balding, vocal cord lengthening, heightened irritability and aggression, and increased libido [5,8]. Furthermore, tissue overgrowth within the cardiovascular system results in left ventricular hypertrophy, congestive heart failure, an increased risk of thrombotic events, and even myocardial infarction. Endocrine dysregulation commonly leads to testicular atrophy, infertility, gynecomastia, prostatic hypertrophy, and menstrual cycle irregularities [5,8]. Elevated liver function tests, denoted by pathologic increases in AST and ALT, are common in individuals, and associated liver damage has been shown to be more pronounced in chronic AAS users [8]. Ancillary compounds, when taken alongside AAS, can help mitigate these systemic side effects; however, contamination of the compounds remains a large concern for users due to the lack of FDA regulation. When produced illicitly, both AAS and ancillary compounds have a high potential to be mislabeled, mis-dosed, and contaminated with heavy metals such as tin, lead, and/or arsenic [9].

Selective androgen receptor modulators

Selective androgen receptor modulators (SARMs) are a class of PEDs that differ from AAS regarding their tissue specificity. SARMs were originally engineered with the purpose of inducing muscle growth and improving bone density while avoiding the traditional virilizing effects associated with AAS use [10]. SARMs accomplish this by acting as selective agonists on androgen receptors within skeletal muscle cells and osteoblasts exclusively, avoiding excess androgenic activation within the testes and prostate [11]. These ligands enter the cell akin to most other steroids: diffusing passively across the plasma membrane, binding to an unoccupied AR in the cytoplasm and displacing it from its respective heat shock protein, and experiencing further modification through the binding of co-regulators and co-activators [12]. The exact mechanism by which tissue specificity is achieved is unknown, yet it is proposed that SARMs’ inability to be metabolized by 5-alpha reductase (which produces DHT), as well as aromatases (which convert androgens into estrogens), plays a major role [13]. Moreover, SARMs’ ability to antagonize the N-terminus to C-terminus interaction of the AR, commonly induced by AAS binding, likely aids in the function of activity modulation and thus the specific effect of the AR [13]. Similar to AR activation in AAS use, the bound receptor is transported to the nucleus, where it acts as a transcription factor to activate genes responsible for increased cellular proliferation, protein synthesis, and associated hypertrophy [12].

SARMs find particular favor amongst users attempting to avoid the systemic and androgenic consequences of AAS. Even so, SARMs are not without their own adverse effects. The three most common self-reported side effects of SARM use are decreased testicular size, increased presence of acne, and uncontrolled mood swings [14]. This side-effect profile indicates that SARMs still invoke effects outside of the intended tissue by altering the hypothalamic-pituitary-adrenal (HPA) axis, a system involved in widespread hormonal regulation of the endocrine system.

Growth hormones and growth hormone secretagogues

GH is produced and secreted from somatotrophs within the anterior pituitary gland. GH functions through both direct action on biological receptors as well as induction of insulin-like growth factor-1 (IGF-1) synthesis and release mainly from liver hepatocytes [15]. IGF-1 mediates the majority of GH’s physiological function through MAP kinase and PI3 kinase/AKT signaling and serves as a means to increase the generation of lean muscle mass, decrease adiposity through increasing lipolysis, and increase differentiation and proliferation of stem cells within both muscle and bone. The end result is noted as cellular hypertrophy and hyperplasia in various organ tissues [16]. This is concerning because aberrant, excessive activation of the P13K pathway has been suspected to induce neoplastic/dysplastic changes within various tissues. For this reason, P13K inhibitors have recently emerged as promising treatments for various oncological disorders, including breast, ovarian, and prostatic malignancies [17]. The specific effects of endocrine (systemically circulating) and paracrine (locally produced) IGF-1 are not fully understood, yet it appears that both analogs play a role in the activation of satellite cells. These satellite cells, following activation, then fuse with myofibrils and decrease mitochondrial apoptosis to allow for muscle repair [18]. Secretagogues of GH, known as GH-related peptides, exert similar biological functions as their endogenous counterparts and are easily synthesized through recombinant DNA methods. Despite the ease of synthesis, the use of these compounds is complicated by either low oral bioavailability or short half-lives following injection [15].

GH utilization, specifically within the athletic community, has occurred for decades due to the compound’s ability to stimulate muscular hypertrophy while simultaneously breaking down adipose tissue. Nonetheless, numerous adverse effects have been documented following repeated use of the compound and related secretagogues. Fluid retention is commonly observed, which manifests as arthralgias (commonly, carpal tunnel syndrome) or excess cerebrospinal fluid (pseudotumor cerebri) [19]. Further unintended consequences involve developing insulin resistance from supraphysiological quantities of circulating GH, progressing to disorders such as type 2 diabetes mellitus, hypertension, glucose intolerance, hemorrhagic strokes, and various malignancies [19]. The development of malignant neoplasms is of particular concern due to the effect of GHs on cellular replication. IGF-1 is often overexpressed in cancers, leading to an increased rate of tumor progression and inhibition of protective cellular apoptosis [20]. Although the compound itself does not induce malignancy, GH modulates cells to undergo replication at an accelerated rate. Thus, various DNA replicative mechanisms are more prone to committing errors and have decreased time to repair these respective errors. When amplified across multiple replication cycles, these errors can reach the critical point of impairing normal cellular processes. Excess cellular hyperplasia, one hallmark of a neoplastic process, is likely to occur in any tissue that experiences these chronic replicative errors within genes responsible for restricting pathological growth (i.e., tumor suppressor genes) [21].

Aromatase inhibitors

Aromatase serves as the main mechanism of estradiol production within the body. This enzyme exerts its effect by converting testosterone to estradiol, the most potent form of the hormone estrogen, and androstenedione to estrone [22]. As it relates to hormonal regulation, the level of circulating estradiol contributes to negative-feedback mechanisms within the hypothalamus to decrease the secretion of gonadotropin-releasing hormone (GnRH). GnRH is responsible for inducing the release of follicle-stimulating hormone (FSH) and luteinizing hormone (LH) from the anterior pituitary gland, with the release of FSH and LH then stimulating estrogen and testosterone production in gonadal structures and hence completing the HPA axis [22]. AIs find common use in medicine as a means of therapeutic treatment for estrogen receptor-positive breast cancers, a subset of breast cancer that experiences accelerated growth in the presence of estrogen. Inhibitors are effective treatment modalities, as this subset of breast cancer overexpresses the aromatase enzyme [23]. Off-label users utilizing AIs as ancillary compounds with PEDs do so in an effort to reduce circulating estradiol levels, thus inhibiting the negative feedback mechanism intended to restrict excess testosterone release. Similarly, the use of AIs slows the enzyme responsible for the degradation of testosterone itself [24]. Since many PEDs achieve an intended physiologic use case through elevating testosterone levels, this consequently leads to increased conversion to estradiol via aromatase. Consequently, AIs serve a role in minimizing the side effects related to heightened estradiol in men, including decreased libido, erectile dysfunction, gynecomastia, and fatigue [25].

Selective estrogen receptor modulators

SERMs are orally effective, non-steroidal analogs of estradiol with mixed agonist-antagonist effects on estrogen receptors. SERMs act as ligands for these receptors, displacing estradiol and preventing the subsequent signaling in a particular tissue cell type [26]. Due to slight structural differences in estrogen receptors throughout different cell types, each SERM can be constructed to preferentially stabilize a specific receptor subset, thus acting as an agonist in some tissues and an antagonist in others [27]. In a clinical setting, SERMs often take advantage of estradiol’s osteogenic effects in order to treat osteoporosis. Additionally, SERMs are used in breast cancer treatment for properties related to receptor antagonism, serving as a modulator to inhibit erroneous tumor growth in estrogen-receptive malignancies [27]. Off-label users utilize SERMs in a manner similar to AIs, intending to mitigate side effects perpetuated by excess estradiol. Many SERMs, however, provide the additional benefit of strengthening osseous tissue through estradiol agonist action [24].

Ancillary compounds (finasteride and Propecia)

In this study, finasteride and Propecia comprise the entirety of the ancillary compounds; however, ancillary compounds were loosely defined as substances commonly taken with PED to mitigate anticipated side effects, such as androgenetic alopecia (male pattern baldness) [28]. Propecia, the brand name for finasteride, is available in both oral and topical dosages and finds clinical use in treating androgenetic alopecia by competitively inhibiting the 5-alpha-reductase enzyme [29,30]. The pathogenesis of androgenetic alopecia is hastened by the binding of DHT to ARs located within the dermis papillae at the base of the hair follicle, initiating changes in follicular activity [31]. Since DHT is produced by the conversion of testosterone using a 5-alpha-reductase, competitive inhibition of 5-alpha-reductase via finasteride use is a favorable option for slowing or preventing the progression of male pattern baldness [30]. PED use often accelerates hair loss in users prone to male pattern baldness; therefore, it is favorable to analyze related trends in Propecia and finasteride to address potential hair loss and its respective effects on quality of life [32].

## Materials and methods

Google Trends data

All trend data was collected using GT, with each drug queried over a defined temporal window across the United States (US); all classes, excluding SARMs, were examined from June 26, 2016, to June 20, 2021, with SARMs being analyzed during a separate, smaller window (January 1, 2019, to June 20, 2021, due to an anomalous single-week spike in RSV, which made the aggregate data meaningless. After each search, the temporal and regional data were downloaded as comma-separated values, systematically stored, and analyzed.

Categorization of search terms

All trend data was collected using GT and allocated into distinct PED categories: AAS, SARMs, GHs, and ancillary compounds, including PCTs, SERMs, and AIs. RSV was aggregated over a five-year temporal window for each class of PED, excluding SARMs, being that various drugs in this class experienced an abnormal, momentous two-week spike in search volume, which impeded accurate analysis; data on SARMs was instead collected from 1/1/2019 to 6/20/2021. Orally ingestible and injectable AAS drugs were sorted and analyzed separately. All compounds within the ancillary category were analyzed as a single class.

Syntax analysis

Variations in syntax popularity were crucial to determining population-wide interest in PEDs; thus, the RSV of each branded drug was first compared to its generic name over the five-year temporal window, providing insight into trends in brand recognition and interest. For compounds lacking a branded name, the compound was analyzed without such comparison. Following data aggregation for each individual drug, the highest average RSV within each category was determined and subsequently utilized to examine trends across PEDs as a whole, with each leading RSV term compared against others in the separate classes. Further syntax analysis was conducted for synonymous nomenclature regarding each class (i.e., the search query “roids'' was compared to the search query “steroids”). Non-standard nomenclature was aggregated by scrubbing popular Reddit pages: r/bodybuilding, r/Steroids, and r/MorePlatesMoreDates. The leading respective class designations were compared against one another. The acronyms “AAS” and “PCT” were avoided during syntax comparison due to the vast degree of use in subject matter outside of PEDs.

Statistical analysis

All data was collected from GT queries and subsequently transferred to Graphpad Prism version 9.3 (GraphPad Software, San Diego, CA). RSV was plotted over a five-year time interval using the software. Statistical tests were conducted to evaluate short-term and long-term variations in data, classifying these changes as random, stationary, or trending in a certain direction. T-tests were utilized to analyze differences between brand and generic names, with the implementation of one-way ANOVA tests in instances where three names were included for a single drug. Pearson’s r was conducted to evaluate any potential associations between the search terms, assigned an r-value ranging from -1 to 1 (i.e., 1 would indicate an exact positive correlation; -1 would indicate an exact negative correlation). All figures and schematics used are original and were generated on BioRender (BioRender, Toronto, Canada).

## Results

Sensitivity syntax

When utilizing a tool like GT, it is vital to adequately contextualize searches to ensure the respective search volume is relative to the desired topic. Google incorporates a “search topic” function, which aggregates search volume for the given, desired phrase in a similar context. Sensitivity syntax analysis reveals which representative phrases - in this case, either the generic name, brand name, single most popular slang term on the aforementioned Reddit forums, or International Union of Pure and Applied Chemistry (IUPAC) nomenclature - for a PED experienced the highest quantity of searches. RSV of generic, brand, and slang names for oral AAS revealed Anadrol (oxymetholone), Dbol (Dianabol, methandrostenolone), Anavar (oxandrolone), Halotestin (fluoxymesterone), oxandrolone (Oxandrin), Superdrol (methasterone), and Winstrol (winny, stanozolol) were the dominant syntax (highest average RSV) used to search for each of the investigated oral AAS. An identical analysis for syntax was carried out for injectable AAS, with nandrolone decanoate (Deca-Durabolin), Masteron (drostanolone propionate), Durabolin (nandrolone phenylpropionate), Sustanon (testosterone isocaproate), testosterone cypionate (depo-testosterone, test c), trestolone (7-alpha methyl-19-nortestosterone), equipoise (boldenone undecylenate), Primobolan (methenolone enanthate), Tren (Revalor, trenbolone), and DHB (dihydroboldenone cypionate) emerging as the dominant syntax.

Similarly, syntax analysis for four different SARMs was carried out. Though many SARMs exist, we focused our analysis on four main SARMs (Cardarine/Endurobol/GW-501516, Ligandrol/LGD-4033, Testolone/RAD-140, ostarine/Enobosarm/MK-2886, Andarine/S-4) readily available for purchase OTC or from third-party vendors. Interestingly, each SARM displayed preferential syntax. Cardarine, Ligandrol, RAD140, ostarine, and Andarine were the dominant syntax used to search for each SARM. Similarly, the dominant syntax was identified for GHs, with MK-677 (Ibutamoren), IGF-1, and human growth hormone (HGH) having the dominant syntax for each compound. Finally, SERMs, AIs, and ancillary compounds were analyzed. Tamoxifen (Nolvadex), Clomid (clomiphene), letrozole (Femara), cabergoline (Dostinex), anastrozole (Arimidex), and finasteride (Propecia) all had significantly different RSVs for the varying syntax. For the remainder of the analyses, the dominant syntax identified for each compound was used. For example, when comparing SARMs, the terms Ligandrol and RAD-140 would be searched relative to one another since these captured most of the search volume for each compound.

Relative intraclass popularity

**Figure 1:**
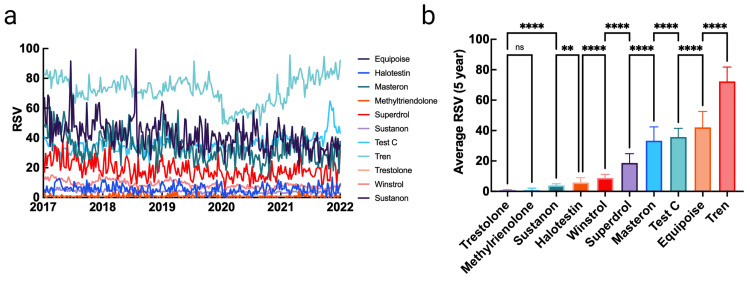
Interest data for anabolic steroids and their relative differences over a five-year period RSV: relative search volume; ns: not significant (p > 0.05); ** p<0.01; **** p<0.0001 Data source: Google Trends

After determining each compound’s most-searched nomenclature, an intraclass analysis was carried out to determine which compound experienced the greatest search volume. To this end, a query comparing each compound’s highest-searched nomenclature was conducted and analyzed. Within the AAS group, the most popular search terms in decreasing order and relative to one another were: Tren (72.28), equipoise (42.10), test c (35.77), Masteron (33.32), Superdrol (18.70), Winstrol (8.79), Halotestin (5.80), Sustanon (3.88), trestolone (0.98), and methyltrienolone (0.92). Uniquely, all AAS saw a decrease in search volume over the five-year period except test c. Additionally, search volume for Tren saw a trend reversal from steadily decreasing to rapidly increasing beginning in 2020, which coincides with the emergence of COVID-19. Significant differences were present between each compound except between trestolone and methyltrienolone, the two least searched compounds. As a class, interest in AAS has decreased over the last five years (Figure 1).

**Figure 2:**
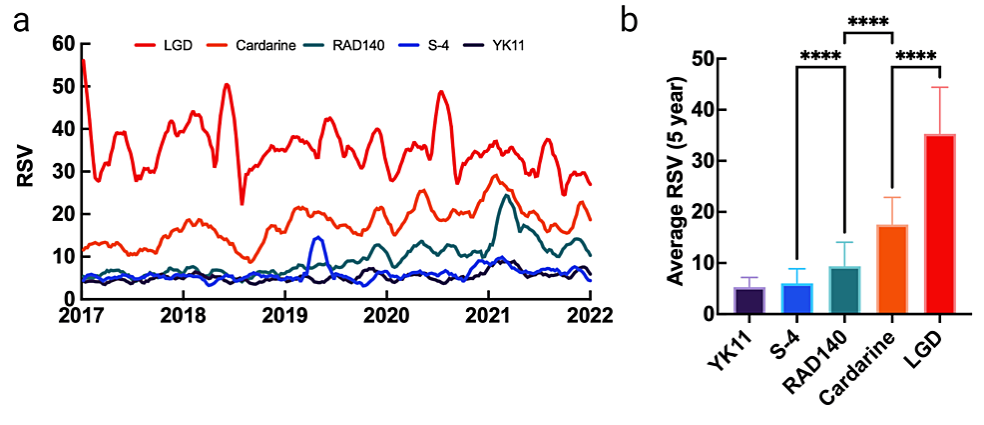
Interest data for selective androgen receptor modulators and their relative differences over a five-year period RSV: relative search volume; **** p<0.0001 Data source: Google Trends

In contrast, each SARM analyzed besides LGD showed a significant increase over the five-year period. The most searched SARMs in decreasing order were LGD (35.30), Cardarine (17.55), RAD140 (9.35), S-4 (6.02), and YK11 (5.28). It is worth noting that ostarine was omitted from this relative intraclass analysis due to a large spike in RSV occurring in late December 2018, which reduced the RSV of the other SARMs to approximately zero. A separate temporal analysis of ostarine is included in the supporting information. Significant differences were observed between each SARM except between YK11 and S-4 (Figure 2).

**Figure 3:**
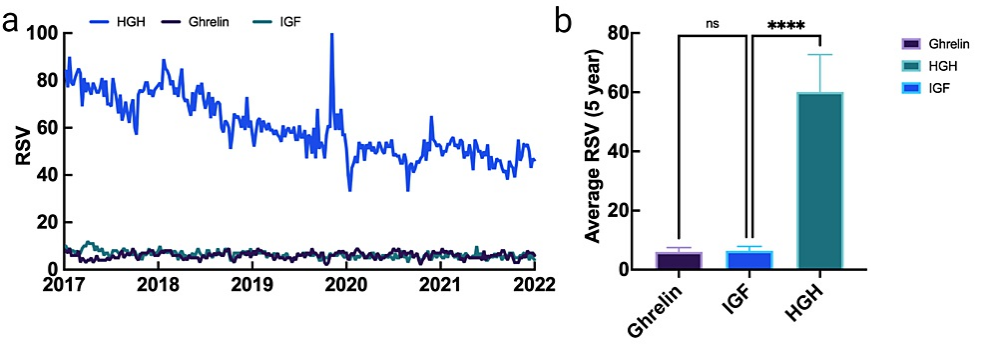
Interest data for grow hormones and their relative differences over a five-year period RSV: relative search volume; HGH: human growth hormone; IGF: insulin-like growth factor; ns: not significant (p>0.05); **** p<0.0001 Data source: Google Trends

An analysis of growth hormones revealed that HGH (60.10) was by far the most searched GH, followed by IGF (6.39) and ghrelin (6.00). Of note, interest in HGH decreased by nearly half over the five-year analysis, while interest in IGF and ghrelin remained largely unchanged (Figure 3).

Relative between-class popularity

**Figure 4:**
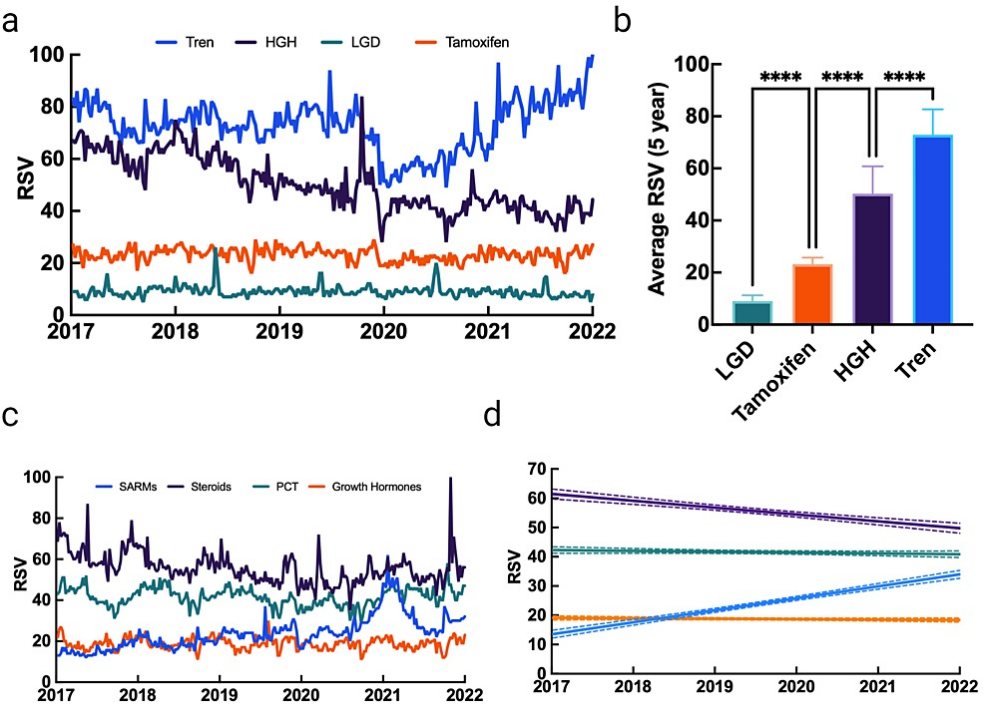
Interest data for grow hormones and their relative differences over a five-year period RSV: relative search volume; HGH: human growth hormone; **** p<0.0001 Data source: Google Trends

After determining which compounds were the most searched within each class, an inter-class analysis comparing the most popular individual compounds was conducted, followed by an analysis comparing each drug class. The compound-specific analysis revealed that Tren (72.87) was the most searched compound, followed by HGH (50.27), tamoxifen (23.18), and LGD (9.00). Interestingly, an analysis comparing SARMs, steroids, PCT, and growth hormones revealed that steroids (55.60) were the most searched, followed by PCT (41.58), SARMs (23.77), and growth hormones (18.73), respectively. However, despite the overall five-year aggregate values, some important trends were observed. First, interest in steroids has largely decreased over the last five years, though a large spike in interest was observed in late 2021. Next, an increase in SARM search volume was observed, while slight decreases in PCT and growth hormones were noted. Taken together, this trend suggests that SARMs may be an emerging class of PEDs that warrant extra clinical attention. Additionally, the increase in SARMs does not correlate to an increase in PCT, perhaps due to the popular misconception that SARMs are largely side-effect-free and would not require PCT, despite evidence obtained through both surveys and in vivo studies (Figure 4).

## Discussion

Key findings

The trends in PEDs and related compounds are vital to a provider’s ability to holistically care for their patient, serving as invaluable insight into their overarching pharmaceutical use.

Clinical observation

From my clinical experience, medically underserved groups in particular seem to be the most affected by unregulated PED use. It appears that, clinically, individuals of all ages and genders are seeking out PED utilization at an increasing rate, typically noting a pressure for both cosmetic and athletic self-improvement as motivating factors. Many of these same individuals suffer from healthcare hesitancy, especially the young-adult age group. They are often afraid to seek out help from a healthcare provider and tend to lack an established, trusting relationship with a provider. Although some young individuals do attempt to seek out medical therapy as they recover from PED or ancillary compound use, they are frequently met with provider unwillingness to offer medical oversight. A recent FDA brief posted on October 31, 2017, which warned about the use of SARMs in bodybuilding, was a welcomed step towards increasing general awareness by specifically mentioning risks such as heart attack, stroke, and liver damage [33]. Being that teenagers are one of the highest-risk groups to utilize these compounds, I would personally lean toward the inclusion of information about prolonged suppression of endogenous hormone production in the setting of PED use. Specifically, it should be explained to any youth contemplating engaging in the use of PEDs that androgen production is vital to the development of secondary sexual characteristics in both males and females. Moreover, the healthcare provider responsible for overseeing an individual engaging in PED use should possess the knowledge to explain all pertinent risks, as well as feel prepared to suggest and encourage pertinent laboratory testing for comorbid pathologies such as hyperlipidemia, sex hormone binding globulin suppression, and hepatotoxicity.

It should be noted that in 2008, over four million males used anabolic androgenic steroids, comprising nearly 2.5% of the American male population [34]. Additionally, when compared to hypogonadal men over the age of 50, hypogonadal men under the age of 50 are more than 10 times as likely to have prior exogenous androgen exposure [32,34]. We, as clinicians, propose that a specific social history should be taken in regard to PED usage. When a patient under the age of 50 asks about their “testosterone” or “hormone” levels, further history of PED use should be obtained using open-ended questions. Asking about the use of PEDs and ancillary compounds during the social history portion of the interview is a good first step. Anecdotally, many patients have a desire to volunteer this information once they have been assured that their provider wants to help them and is willing to do so. We have witnessed first-hand the challenges that arise when underserved patients do not receive the education and clinical expertise they require in these particular situations, and as such, we have developed a true passion for providing the best care possible for this population. We hope this study provides actionable steps for our provider-peers to answer the call as well.

A call to action: improving clinical education surrounding PED use

An understanding of these trends regarding PEDs and related compounds is vital to being able to provide holistic care for patients, namely because patients who use PEDs are walking into clinics across the United States and frequently being seen by healthcare providers. Public health professionals have undertaken large initiatives in the last few decades to improve identification and subsequently modify the attitudes of various substance users, finding particular success in their nicotine and alcohol use campaigns [35,36]. While the users of nicotine and alcohol may not fit the exact socioeconomic and demographic picture of the users of PEDs, it is clear that a provider can provide a higher quality of care by remaining aware of the latest trends in substance use and speaking with their patients directly. In my practice, using a non-judgmental approach is vital to the development of patient-physician rapport and the willingness of a patient to volunteer helpful subjective information. It also serves to alleviate patient anxiety and reassure the patient that the provider has their best interests at heart. There is a stark difference between condoning use and being non-judgmental. The stigma for any substance use, whether it is marijuana use, alcohol use, or PED use, is a significant barrier to care. The results of this study demonstrate a strong upward trend in the popularity of many PEDs; thus, providers should become accustomed to having conversations with their patients about PED use in addition to other substances.

As indicated by the results of our study, SARMs are well-known in the general community, particularly among younger patients. Nonetheless, many misconceptions surrounding the safety and withdrawal of these substances are propagated across online fitness media outlets. The incongruency in education is exacerbated by highly variable prescribing practices among physicians, especially for compounds such as SERMs and testosterone. For example, consider an 18-year-old patient who has utilized a fitness supplement that, knowingly or unknowingly, contained SARMs. This patient may present with hypogonadal symptoms and low FSH/LH or testosterone and could theoretically recover hypothalamic-pituitary-gonadal function quickly with a short course of a SERM. In another example, a patient may present after utilizing a 19-nor testosterone ester like nandrolone decanoate, a compound known for its robust suppressive progestogenic activity even after the passage of numerous half-life durations. A provider who is not adeptly trained in the clinically applicable pharmacokinetics of these compounds could easily prescribe either individual testosterone replacement therapy. In actuality, with good medical oversight, both patients are likely to recover using their own endogenous androgenic hormone production and avoid the deleterious, possibly permanent effects of further exogenous supplementation. We frequently encounter individuals who state they are “doomed to remain on testosterone replacement therapy forever." Furthermore, lab values can be preemptively anticipated, and unnecessary imaging foregone if a provider has knowledge about the likely concomitant hyperprolactinemia and hypercortisolism resulting after the course of nandrolone. During withdrawal, these conditions are common and should be evaluated and trended back to normal. This would be similar to the evaluation for hepatic damage, arrhythmia, and varices associated with alcohol withdrawal. Harm reduction strategies for PED use, especially anabolic steroids, have already been discussed in prior literature and are simply awaiting utilization [37]. It is time to bridge the fundamental gap in this field of knowledge, one that can significantly hinder the care of a recovering patient and possibly result in irreparable damage.

Limitations

There are several key limitations to this study design that warrant mentioning. First, our analysis relies heavily upon search interest in specific terms, which may not capture the entire interest in the related subject (i.e., searching for “anabolic steroids” and “anabolic androgenic steroids” results in different RSVs). While we attempted to mitigate this limitation using a syntax analysis, the cumulative interest in a given field is only represented by the selected search terms rather than captured in their entirety. Another significant limitation is the inability to express RSV in raw searches. To this end, singular catalyzing events (i.e., large news reports, etc.) drive RSV to unexpected heights, creating large spikes in RSV and minimizing long-term trends. Future studies utilizing a different search engine or another social media platform may address this drawback.

In addition to the variations associated with RSV and capturing interest in a subject, GT studies are unable to contextualize searches. In other words, the only way to contextualize searches is by analyzing related queries. Therefore, we are unable to determine the demographics of the curated users or their respective intentions. While this data would allow us to further determine who is searching for these terms and the motivations behind their actions, the raw data presented here is to be taken as a representative summary. Further, Google does not serve as the sole search engine for the population and may possess a specific set of demographic characteristics and PED usage that differs from that of other search engines and their respective users. Finally, clinical observations are anecdotal and suffer from limitations associated with single-provider insight.

## Conclusions

In this study, for the first time, the use of social data to identify trends in PEDs and related compound use was introduced. In addition to using social data as a proxy for monitoring interest in PEDs, this study delved into the similarities, differences, backgrounds, potential complications, and popular on/off-label uses relative to performance enhancement. Elucidating how different substances are used synergistically in a performance-enhancing context allows the subject to evaluate relative compound usage. For example, it is apparent that interest in PCT does not match the explosive interest in RAD140 over the last five years. Identifying these types of discrepancies allows healthcare providers to address common misconceptions (i.e., the notion that SARMs do not require hormone surveillance or prescription medication to assist recovery) and further educate patients in a clinical setting. Additionally, emerging trends were identified regarding PEDs, both within and between drug groups. Interest in SARMs has risen significantly over the past several years, while interest in AAS has remained consistent and, in some cases, even decreased. The identification of these key trends allows providers to tailor practices around emerging compounds to best address, treat, and educate patients in a clinical setting. Finally, we discuss example cases of SARM and steroid use and how knowledge of sequelae can lead to better evidence-based medicine. The hope is that this study empowers other providers to provide patient-centered care and engage in further PED use studies as trends continue to change with time. Furthermore, by presenting a methodical approach to analyzing readily available GT data, the hope is that providers can utilize this tool to assist with trend recognition within the specific locations that comprise the provider’s respective patient population.
